# A Temporal Window Attention-Based Window-Dependent Long Short-Term Memory Network for Multivariate Time Series Prediction

**DOI:** 10.3390/e25010010

**Published:** 2022-12-21

**Authors:** Shuang Han, Hongbin Dong

**Affiliations:** College of Computer Science and Technology, Harbin Engineering University, Harbin 150001, China

**Keywords:** multivariate time series, temporal window attention, encoder–decoder, LSTM

## Abstract

Multivariate time series prediction models perform the required operation on a specific window length of a given input. However, capturing complex and nonlinear interdependencies in each temporal window remains challenging. The typical attention mechanisms assign a weight for a variable at the same time or the features of each previous time step to capture spatio-temporal correlations. However, it fails to directly extract each time step’s relevant features that affect future values to learn the spatio-temporal pattern from a global perspective. To this end, a temporal window attention-based window-dependent long short-term memory network (TWA-WDLSTM) is proposed to enhance the temporal dependencies, which exploits the encoder–decoder framework. In the encoder, we design a temporal window attention mechanism to select relevant exogenous series in a temporal window. Furthermore, we introduce a window-dependent long short-term memory network (WDLSTM) to encode the input sequences in a temporal window into a feature representation and capture very long term dependencies. In the decoder, we use WDLSTM to generate the prediction values. We applied our model to four real-world datasets in comparison to a variety of state-of-the-art models. The experimental results suggest that TWA-WDLSTM can outperform comparison models. In addition, the temporal window attention mechanism has good interpretability. We can observe which variable contributes to the future value.

## 1. Introduction

Predicting the multivariate time series precisely is of great significance in real life, such as traffic prediction, virus spread prediction, energy prediction, and air quality prediction [1]. For instance, policymakers can plan interventions by predicting epidemic trajectories [2], and people make outdoor activity planning by predicting future air quality [3]. For multivariate time series prediction, this is a common practice that the temporal window size is required to divide the time series data into multiple feature matrices with fixed dimensions [4] as the input. That is to say that there is no available information before the window. However, in practice, it is hard to contain all useful information in an appropriate window size. Therefore, considering the information of the previous window can help to find meaningful insights into time series data.

**Figure 1:**
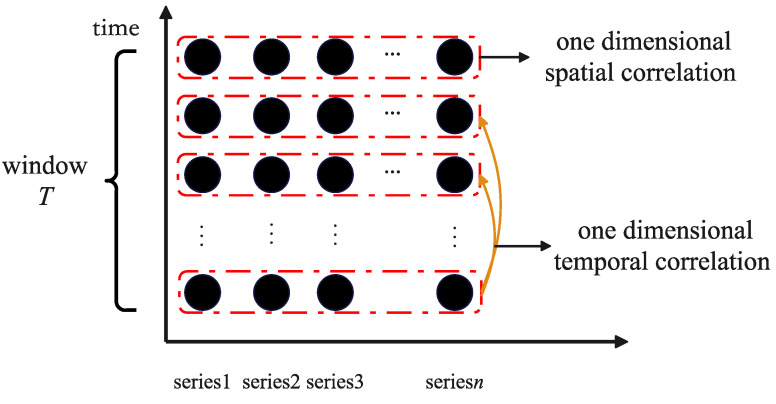
One dimensional spatio-temporal correlation. A black circle represents a variable.

Capturing spatio-temporal correlation is the main problem in multivariate time series prediction tasks. The spatio-temporal correlations within a temporal window represent the influence of different exogenous series on prediction at different time steps. Most studies capture one-dimensional spatio-temporal correlations, such as the spatial correlations among variables at the same time step and temporal correlation among different time steps, as shown in Figure 1. The one-dimensional spatio-temporal correlations focus only on locally relevant variables, which leads to ignoring some important information. For instance, although variable 1 at time *t* is more important than variable 2 at time *i*, the weight coefficient of variable 1 is smaller than that of variable 2. Therefore, it is necessary to consider the global information (i.e., two-dimensional spatio-temporal correlations) within a temporal window for multivariate time series prediction.

Historically, a vital issue of time series prediction is capturing and exploiting the dynamic temporal dependencies in a temporal window [5]. The autoregressive integrated moving average (ARIMA) [6,7] focuses only on capturing the temporal dependence of the target series itself and ignores the exogenous series. Hence, ARIMA is not suitable for modeling dynamic relationships among variables for multivariate time series. The vector autoregressive (VAR) [8] model is developed to utilize exogenous series features. The hidden Markov model (HMM) is a classic statistical model that contributes to extracting the dynamic temporal behavior of the multivariate time series [9]. However, VAR and HMM suffer from the problem of overfitting with large-scale time series. To overcome this limitation, recurrent neural network (RNN) and its variants (e.g., long short-term memory network (LSTM) [10] and gated recurrent unit (GRU) [11]) have been developed for multivariate time series prediction. Nevertheless, they fail to differentiate the contribution of the exogenous series to the target series, resulting in limited information.

Most single time series prediction networks are no longer sufficient for fitting a given multivariate time series due to the complexity and variability of time series structure [9,12]. Encoder–decoder networks based on LSTM or GRU units are popular due to their ability to handle complex time series. However, they are limited in capturing the dependencies of the long input sequence. Inspired by the learning theory of human attention, attention-based encoder–decoder networks have been applied to select the most relevant series. For instance, the two-stage attention mechanism [13] has been designed to predict multivariate time series, which employ input attention to select the exogenous series and temporal attention to capture historical information. Subsequently, the multistage attention mechanism [14] has been developed to capture the influence information from different time stages. Recently, the temporal self-attention mechanism [1] has been introduced into the encoder–decoder to extract the temporal dependence. These attention-based encoder–decoder networks achieve state-of-the-art performance by extracting the spatial–temporal dependence for multivariate time series prediction. Nevertheless, these models focus on important information in spatial and temporal dimensions, respectively. In addition, LSTM and GRU summarize the information only within a temporal window so that they cannot memorize very long term information.

To address these issues, we proposed a temporal window attention-based window-dependent long short-term memory network (TWA-WDLSTM) to predict future values. The contributions of our work are summarized as follows:

(1) Temporal window attention mechanism. We develop a new attention concept, namely a temporal window attention mechanism, to learn the spatio-temporal pattern. The temporal window attention mechanism learns the weight distribution strategy to select the relevant variables in a temporal window; hence, it can capture two-dimensional spatio-temporal correlations from a global perspective.
(2) Window-dependent long short-term memory network (WDLSTM). We design a novel recurrent neural network as the encoder and decoder to enforce the long-term temporal dependencies among time series because it utilizes the previous temporal window hidden state matrix.
(3) Real evaluation. We construct extensive experiments on four real-world datasets to demonstrate the effectiveness of our model. The results show that our model outperforms state-of-the-art comparison models.

The remainder of this paper is organized as follows. We provide a literature review on time series prediction methods in Section 2. We define the notations and problem formulation in Section 3. We introduce the specific details of our model in Section 4. We design experiments to test the validity of our model in different fields and analyse the experimental results in Section 5. We summarize our work and future work in Section 5.

## 2. Related works

### 2.1. Recurrent Neural Network

As deep learning develops, more models for multivariate time series prediction have been proposed. The recurrent neural network (RNN) with the capacity to gain short-term dependencies of time series becomes one of the most outstanding multivariate time series prediction models. Recently, some advanced RNN variants were proposed to overcome vanishing gradient and capture long-term dependencies. For instance, Feng et al. [15] introduced the clockwork RNN, which runs the hidden layer at different clock speeds to solve the long-term dependency problem. Zhang et al. [16] modified the GRU architectures, in which gates explicitly regulate two distinct types of memories to predict medical records and multi-frequency phonetic time series. Ma et al. [17] designed temporal pyramid RNN to gain long-term and multi-scale temporal dependencies. Harutyunyan et al. [18] introduced a modification of LSTM, namely, channel-wise LSTM, which is most remarkable in length-of-stay prediction. Zhang et al. [19] designed CloudLSTM, which employs a dynamic point-cloud convolution operator as the core component for spatial–temporal point-cloud stream forecasting. The above RNN methods focus on utilizing the information within a temporal window. Limited by the temporal window size, they cannot fully exploit very long term temporal dependencies.

The input length limits the performance of an RNN, and as the length increases, its ability to extract time features decreases. To address this issue, combining the attention mechanism and RNN to select relevant features provides excellent results. For instance, Wang et al. [20] used two stacked LSTM layers with two attention layers to improve prediction performance. Liu et al. [21] introduced the dual-stage two-phase attention-based LSTM to enhance spatial features and discover long-term dependencies to predict long-term time series. Huang et al. [3] designed a spatial attention–embedded LSTM to tackle the intricate spatial–temporal interactions for air quality prediction. Liang et al. [22] developed multi-level attention-based LSTM to capture the complex correlations between variables to achieve geo-sensory time series prediction. Deng et al. [23] employed a graph attention mechanism to learn the dependence relationships for anomaly detection. Preeti et al. [24] used self-attention to focus more on relevant parts of the time series in the spatial dimension. However, these studies pay more attention to designing various attention mechanisms to obtain the relationships in the spatial dimension. In the temporal dimension, the typical temporal attention mechanism is used to select the relevant time steps and ignore the relevant variables.

### 2.2. Convolutional Neural Network

The Convolutional Neural Networks (CNNs) model is popular because of multi-threaded GPU computing. Wang et al. [25] designed multiple CNNs to integrate the correlations among multiple periods for periodic multivariate time series prediction. Wu et al. [26] developed temporal convolution modules to learn temporal dependencies. However, CNNs fail to capture long-term temporal dependencies for multivariate time series prediction. Most studies additionally used other methods to extract global temporal correlations. For instance, Lai et al. [27] employed convolutional neural network models to extract local temporal features of time series and GRU to discover long-term dependencies for time series. Cao et al. [28] introduced a temporal convolutional network to mine short-term temporal correlations and an encoder–decoder attention module to gain long-term temporal correlations for traffic flow prediction.

## 3. Model

### 3.1. Notation and Problem Statement

Multivariate time series data are composed of multiple exogenous series and one target series. Given *N* exogenous series and the temporal window size *T*, we divide the time series data into multiple feature matrices. A matrix with fixed dimensions is as follows:

$$X_{t^{\prime }}=\left(x^{1},\;x^{2},\ldots ,\;x^{N}\right)\;={\left(x_{1},x_{2},\ldots ,x_{T}\right)}^{⊤}\;=\left[\begin{array}{c} \begin{array}{c} x_{1}^{1}\\ \vdots \\ x_{t}^{1}\\ \vdots \end{array}\\ x_{T}^{1} \end{array}\begin{array}{c} \begin{array}{c} x_{1}^{2}\\ \vdots \\ x_{t}^{2}\\ \vdots \end{array}\\ x_{T}^{2} \end{array}\begin{array}{c} \begin{array}{c} \cdots \\ \vdots \\ \cdots \\ \vdots \end{array}\\ \cdots \end{array}\begin{array}{c} \begin{array}{c} x_{1}^{N}\\ \vdots \\ x_{t}^{N}\\ \vdots \end{array}\\ x_{T}^{N} \end{array}\right]$$

where $x_{t}^{i}$ is the i-th exogenous series at time *t*, and $t^{\prime }$ represents the $t^{\prime }$-th temporal window.

Given the previous values of the target series, $y_{t^{\prime }}=(y_{1},y_{2},\ldots ,y_{T})\;$ with $y_{t}\in \mathbb{R}$, and past values of *N* exogenous series, i.e.,$\;X_{t^{\prime }}\in {\mathbb{R}}^{T\times N}$, our aim is to design a model for learning complex nonlinear relationships among time series:

$${\hat{y}}_{t^{\prime },T+1}{=F(y}_{t^{\prime }},\;X_{t^{\prime }})$$

where *F* (·) is a prediction model we aim to construct.

### 3.2. Exhaustive Description of Model

**Figure 2:**
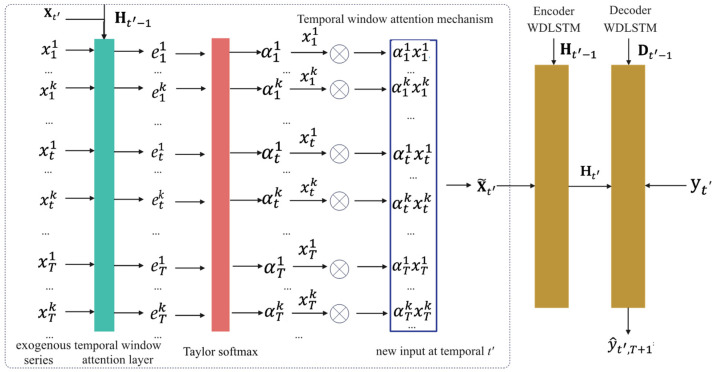
Overall framework of the proposed TWA-WDLSTM model. The temporal window attention mechanism is calculated in the dashed box. The green box denotes the temporal window attention mechanism that computes weight coefficient $\alpha_{t}^{k}$ based on the variable $x_{t}^{k}$, the feature matrix $X_{t^{\prime }}$, and the previously hidden state matrix $H_{t^{\prime }-1}$. The newly computed ${\tilde{X}}_{t^{\prime }}$ is fed into the encoder WDLSTM unit. The encoded hidden state matrix $H_{t^{\prime }}$ is used as an input to the decoder WDLSTM unit and generates the final prediction result ${\hat{y}}_{t^{\prime },T+1}$.

**Figure 3:**
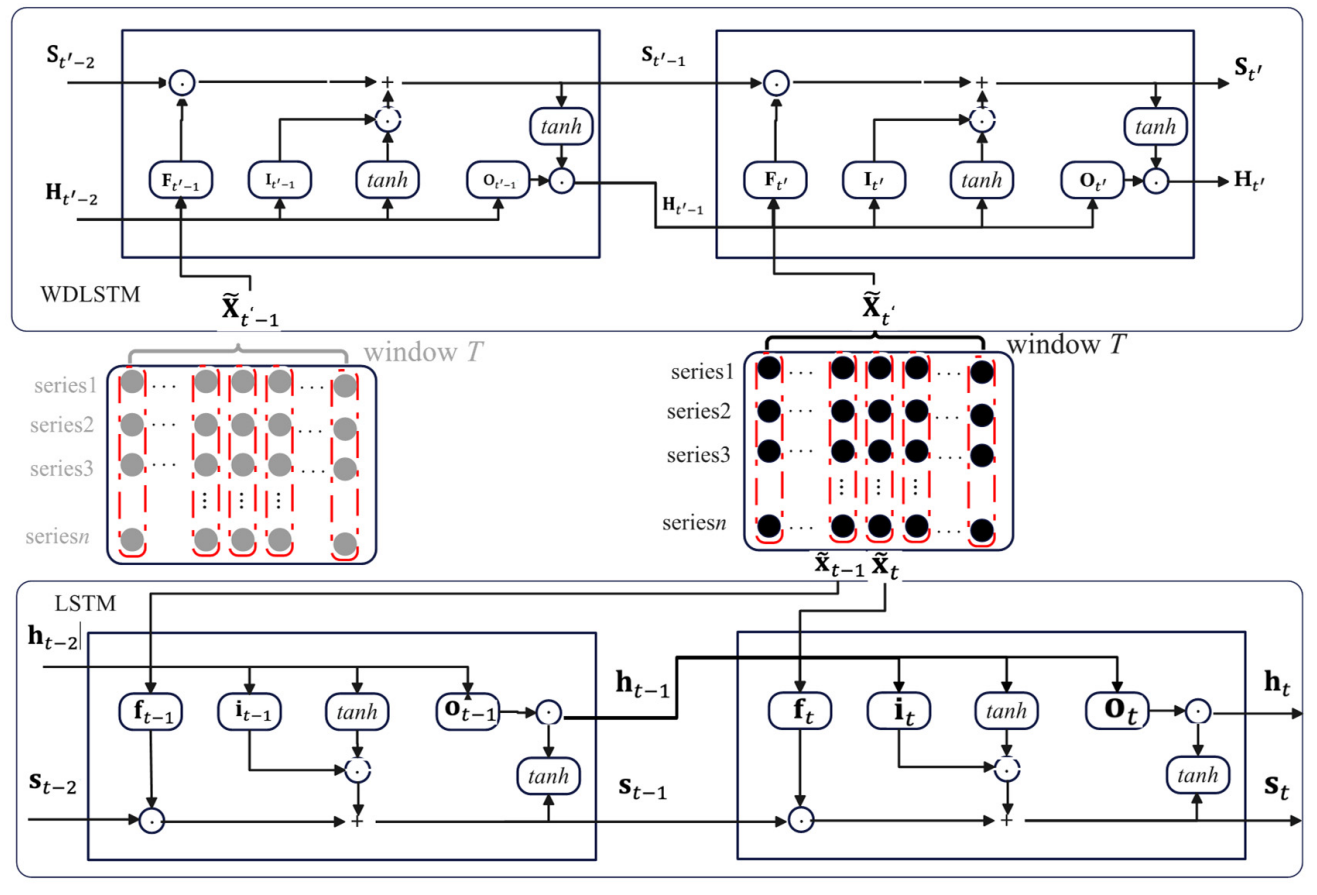
The structure of WDLSTM and LSTM. The black and grey circles represent elements of current input ${\tilde{X}}_{t^{\prime }}$ and previous input ${\tilde{X}}_{t^{\prime }-1}$, respectively. The memory cells $S_{t^{\prime }}$, forget gate $F_{t^{\prime }}$, input gate $I_{t^{\prime }}$, and output gate $O_{t^{\prime }}$ are the components of WDLSTM. The matrix ${\tilde{X}}_{t^{\prime }}$ is fed into WDLSTM to generate hidden state matrix $H_{t^{\prime }}$. The memory cells $s_{t}$, forget gate $f_{t}$, input gate $i_{t}$, and output gate $o_{t}$ are the components of LSTM. ${\tilde{x}}_{t},$ which is the element vector of ${\tilde{X}}_{t^{\prime }}$, is the input of LSTM.

We employ the attention-based encoder–decoder network to perform a multivariate time series prediction, as depicted in Figure 2. Specifically, in the encoder, we propose a temporal window attention mechanism to adaptively select relevant variables in a temporal window. To capture very long temporal dependencies, WDLSTM is introduced to encode the input features into a matrix hidden state. In the decoder, WDLSTM decodes the encoded input features to predict future values. The detailed structure of WDLSTM is shown in Figure 3.

#### 3.2.1. Temporal Window Attention Mechanism

Most existing work focuses mainly on designing different attention mechanisms to select the relevant variables at the same time step or capture historical information at different time steps to improve prediction performance. However, there is a critical defect in applying existing attention mechanisms for multivariate time series prediction because it fails to select essential variables in terms of prediction utility. Moreover, the temporal attention mechanisms perform a weighted average of historical information, failing to detect time steps that are useful for prediction. Ref. [20] has proved that utilizing both temporal and spatial attention can improve prediction accuracy. Hence, we design a temporal window attention mechanism to capture two-dimensional spatio-temporal correlations in a temporal window.

For the prediction task, we use the features within the temporal window to generate value for the next time step. We take the series matrix within a temporal window, $X_{t^{\prime }}=\left(x^{1},\;x^{2},\ldots ,\;x^{N}\right)={\left(x_{1},x_{2},\ldots ,x_{T}\right)}^{⊤}\in {\mathbb{R}}^{T\times N}$ and a variable $x_{t}^{k}\in \mathbb{R}$ as input. We define a temporal window attention mechanism via a multilayer perceptron to score the importance of the k-th input variable at time t within a temporal window, as follows:

(1) $$e_{t}^{k}{=v}_{e}^{⊤}\tan h([H_{t^{\prime }-1};S_{t^{\prime }-1}]W_{e}+X_{t^{\prime }}W_{e}^{\prime }+W_{e}^{\prime \prime }x_{t}^{k}+b_{e})$$

where $[H_{t^{\prime }-1};S_{t^{\prime }-1}]\in {\mathbb{R}}^{T\times 2n}$ is a concatenation operation, $H_{t-1}$ and $S_{t-1}$ are the hidden state and cell state of the WDLSTM unit, respectively, and $v_{e}\in {\mathbb{R}}^{T}$,$\;W_{e}\in {\mathbb{R}}^{2n}$,$\;W_{e}^{\prime }\in {\mathbb{R}}^{N}$, $W_{e}^{\prime \prime }\in {\mathbb{R}}^{T}$, and $b_{e}\in {\mathbb{R}}^{T}$ are parameters to learn.

After that, it is necessary to use the softmax function to normalize the attention weights. The softmax function helps to capture long-term dependencies yet prohibits its scale-up due to the time complexity. To gain more variables to be useful for prediction, we use the Taylor softmax [29] function instead of the softmax function, as follows:

(2) $$\alpha_{t}^{k}=\frac{1+e_{t}^{k}+0.5{\left(e_{t}^{k}\right)}^{2}}{\sum_{t}^{T}\sum_{i}^{N}1+e_{t}^{i}+0.5{\left(e_{t}^{i}\right)}^{2}}$$

where $\alpha_{t}^{k}$ is the attention weight measuring the importance of the *k*-th series to future value at time *t* in temporal window $t^{\prime }$. The Taylor softmax function employs second-order Taylor expansion of the exponential, $exp\;(e_{t}^{k})\approx 1+e_{t}^{k}+0.5{\left(e_{t}^{k}\right)}^{2}$. Moreover, the numerator $1+e_{t}^{k}+0.5{\left(e_{t}^{k}\right)}^{2}$ is positive definite and never becomes zero because its minimum value is 0.5. Hence, the Taylor softmax function can enhance numerical stability. Once we obtain the attention weights, the new input matrix is computed as follows:

$${\tilde{X}}_{t^{\prime }}=\left[\begin{array}{c} \begin{array}{c} \alpha_{1}^{1}x_{1\;}^{1}\\ \vdots \\ \alpha_{t}^{1}x_{t}^{1}\\ \vdots \end{array}\\ \alpha_{T}^{1}x_{T}^{1} \end{array}\;\begin{array}{c} \begin{array}{c} \alpha_{1}^{2}x_{1}^{2}\\ \vdots \\ \alpha_{t}^{2}x_{t}^{2}\\ \vdots \end{array}\\ \alpha_{T}^{2}x_{T}^{2} \end{array}\begin{array}{c} \begin{array}{c} \cdots \\ \vdots \\ \cdots \;\\ \vdots \end{array}\\ \cdots \end{array}\begin{array}{c} \begin{array}{c} \;\alpha_{1}^{n}x_{1}^{N}\\ \vdots \\ \alpha_{t}^{n}x_{t}^{N}\\ \vdots \end{array}\\ \;\alpha_{T}^{3}x_{T}^{N} \end{array}\right]$$

#### 3.2.2. Encoder–Decoder

In both the encoder and decoder, LSTM, which can memorize historical information, is employed to encode/decode the input sequences into high-level feature representations. In practice, LSTM usually fails to memorize very long term dependencies because it only considers the features within a temporal window. We propose a window-dependent long short-term memory network (WDLSTM) to enhance the learning ability of the long-term temporal dependencies. The idea of WDLSTM is to encapsulate the features matrix of a temporal window as a hidden state matrix. As standard LSTM neural networks, WDLSTM has the memory cells $S_{t^{\prime }}$ and the gate control units, such as forget gate $F_{t^{\prime }}$, input gate $I_{t^{\prime }}$, and output gate $O_{t^{\prime }}$. The gate matrices and hidden state matrices of WDLSTM are denoted with uppercase and boldface to differentiate the gate vectors and hidden state vector of LSTM. In the encoder, given the newly computed ${\tilde{X}}_{t^{\prime }}$ at temporal window $t^{\prime }$ and the previous window hidden state matrix $H_{t^{\prime }-1}$, the WDLSTM unit update is summarized as follows:

(3) $$F_{t^{\prime }}=\sigma (\;[H_{t^{\prime }-1};{\tilde{X}}_{t^{\prime }}{]W}_{F}{+b}_{F})$$

(4) $$I_{t^{\prime }}=\sigma ([H_{t^{\prime }-1};{\tilde{X}}_{t^{\prime }}{]W}_{I}{+b}_{I})$$

(5) $$O_{t^{\prime }}=\sigma ([H_{t^{\prime }-1};{\tilde{X}}_{t^{\prime }}{]W}_{O}{+b}_{O})$$

(6) $${\tilde{S}}_{t^{\prime }}\;=\;\tan h(\left[H_{t^{\prime }-1};{\tilde{X}}_{t^{\prime }}]W_{S}+b_{S}\right)$$

(7) $$S_{t^{\prime }}=F_{t^{\prime }}⊙S_{t^{\prime }-1}+I_{t^{\prime }}⊙{\tilde{S}}_{t^{\prime }}$$

(8) $$H_{t^{\prime }}\;\;=\;O_{t^{\prime }}⊙{\tan h(S}_{t^{\prime }})$$

where $[H_{t^{\prime }-1};\tilde{X}]\in {\mathbb{R}}^{T\times \left(n+N\right)}$ is a concatenation of the hidden state matrix $H_{t^{\prime }-1}\in {\mathbb{R}}^{T\times n}$ and the current input matrix ${\tilde{X}}_{t^{\prime }}\;\in {\mathbb{R}}^{T\times N}$, $W_{F}$, $W_{I}$, $W_{O}$, $W_{S}\in {\mathbb{R}}^{\;\left(n+N\right)\times n}$ and $b_{F}$, $b_{I}$, $b_{O}$, $\;b_{S}\in {\mathbb{R}}^{T\times n}$ are parameters to learn. The symbols σ and ⊙ are a logistic sigmoid function and an elementwise multiplication, respectively. Figure 3 presents the structure of the WDLSTM and the difference between WDLSTM and LSTM. The input of WDLSTM is a matrix ${\tilde{X}}_{t^{\prime }}\in {\mathbb{R}}^{T\times N}$ at temporal window $t^{\prime }$, and hidden state matrix $H_{t^{\prime }}\in {\mathbb{R}}^{T\times n}$ is the output of WDLSTM. The input of LSTM is a vector ${\tilde{x}}_{t}\in {\mathbb{R}}^{N}$ at time step t, and hidden state vector $h_{t}\in {\mathbb{R}}^{n}\;$ is the output of LSTM. ${\tilde{x}}_{t}$ is the element vector of ${\tilde{X}}_{t^{\prime }}$. Different from the gate unit of LSTM, that of WDLSTM can control the input and output of information within the temporal window, which changes the value range of the memory, so that the information flows of the temporal window can preserve the very long term dependencies.

In the decoder, we combine the encoder hidden state with the target series $y_{t^{\prime }}$ as the input and then employ the WDLSTM to decode the concatenation. That is to say, the hidden state matrix of the encoder can update the decoder hidden state matrix, which can be defined as follows:

(9) $$D_{t^{\prime }}=f_{MLSTM}\left(D_{t^{\prime }-1},{[H}_{t^{\prime }};y_{t^{\prime }}\right])$$

where $D_{t^{\prime }}\in {\mathbb{R}}^{T\times m}$ is the decoder hidden state matrix, and $f_{MLSTM}$ is a WDLSTM unit. Then $D_{t^{\prime }}$ can be updated as:

(10) $${\ddot{F}}_{t^{\prime }}=\sigma ([D_{t^{\prime }-1}{;\;[H}_{t^{\prime }};y_{t^{\prime }}]]{\ddot{W}}_{F}+{\ddot{b}}_{F})$$

(11) $${\ddot{I}}_{t^{\prime }}=\sigma ([D_{t^{\prime }-1}{;\;[H}_{t^{\prime }};y_{t^{\prime }}]]{\ddot{W}}_{I}+{\ddot{b}}_{I})$$

(12) $${\ddot{O}}_{t^{\prime }}=\sigma ([D_{t^{\prime }-1}{;\;[H}_{t^{\prime }};y_{t^{\prime }}]]{\ddot{W}}_{O}+{\ddot{b}}_{O})$$

(13) $${\tilde{\ddot{S}}}_{t^{\prime }}\;=\;\tan h(\left[D_{t^{\prime }-1};{\;[H}_{t^{\prime }};y_{t^{\prime }}]]{\ddot{W}}_{S}+{\ddot{b}}_{S}\right)$$

(14) $${\ddot{S}}_{t^{\prime }}=F_{t^{\prime }}⊙{\ddot{S}}_{t^{\prime }-1}+I_{t^{\prime }}⊙{\tilde{\ddot{S}}}_{t^{\prime }}$$

(15) $$D_{t^{\prime }}\;=\;{\ddot{O}}_{t^{\prime }}⊙\tan h({\ddot{S}}_{t^{\prime }})$$

where $[D_{t^{\prime }-1}{;\;[H}_{t^{\prime }};y_{t^{\prime }}]]\in {\mathbb{R}}^{T\times \left(m+n+1\right)}$ is a concatenation of the previous hidden state matrix $D_{t^{\prime }-1}$ and the input ${[H}_{t^{\prime }};y_{t^{\prime }}]$. Finally, we employ the linear function to generate the future value:

$${\hat{y}}_{t^{\prime },T+1}=F(y,\;X_{t^{\prime }})\;=v_{y}^{⊤}\left(D_{t^{\prime }}W_{y}+b_{w}\right)+b_{y}$$

where $v_{y}\in {\mathbb{R}}^{T}$, $W_{y}\in {\mathbb{R}}^{m}$, $b_{w}\in {\mathbb{R}}^{T}$, and $b_{y}\in \mathbb{R}$ are parameters to learn.

## 4. Experimental Studies

### 4.1. Datasets

We utilize four real-world time series datasets to evaluate our model. There are missing values in all datasets due to sensor power outages or communication errors. We employ linear interpolation to fill in the missing values. We partition the datasets into the training and test sets by a ratio of 8:2.

1. **Photovoltaic (PVP) power dataset**: The dataset is derived from National Energy Day-ahead PV power, which is competition data. The frequency of data collection is every 15 min. We take the PV power as the target series and choose six relevant features as exogenous series. We select the first 24,000 time steps as the training set and the rest 6000 time steps as the test set.
2. **SML 2010 dataset**: This dataset collected 17 features from the house monitor system. We utilized 16 exogenous series to predict room temperature. We select the first 2211 time steps as the training set and the rest 553 time steps as the test set.
3. **Beijing PM2.5 dataset**: This dataset collected hourly the concentration of PM2.5 and some meteorological readings from air-quality monitoring sites in Beijing, China. The time period is from 1 January 2013 to 31 December 2014, in which the first 14,016 time steps are used to train models and the remaining 3504 time steps are employed to test models.
4. **NASDAQ 100 stock dataset**: The dataset contains the per-minute stock prices of 81 major companies. The NASDAQ 100 is regarded as target series. We select the first 32,448 time steps as the training set and the remaining 8112 time steps as the test set.

### 4.2. Methods for Comparison

We select seven state-of-the-art models as comparison models. The modes are introduced as follows:

**ARIMA** [30]: ARIMA is a typical statistical model for univariate time series prediction. The ARIMA model converts nonstationary time series to stationary data utilizing difference processing.

**LSTM** [31]: LSTM is a widely applied RNN variant designed to mine the long-term temporal dependence hidden in time series.

**DA-RNN** [13]: The attention-based encoder–decoder network for time series prediction employs an input attention mechanism to gain spatial correlations and temporal attention to capture temporal dependencies.

**DSTP-RNN** [15]: The model employs a two-phase attention mechanism to strengthen the spatial correlations and a temporal attention mechanism to capture temporal dependencies for long-term and multivariate time series prediction.

**MTNet** [32]: This trains the nonlinear neural network and autoregressive components in parallel to improve the robustness. The nonlinear neural network uses the memory component to memorize long-term historical information.

**DA-Conv-LSTM** [33]: This exploits the convolutional layer and two-stage attention model to extract the complex spatial-temporal correlation among nearby values.

**CGA-LSTM** [12]: The model employs the correlational attention mechanism to gain the spatial correlation and the graph attention network to learn temporal dependencies.

### 4.3. Parameter and Evaluation Metrics

We execute a grid search strategy and choose the best values for three types of key hyperparameters in our model. All models shared the hyperparameters that are listed in Table 1 for a fair comparison. For the number of time windows *T*, we set *T* ∈ {6, 12, 24, 48} for the PV power dataset that is periodic and *T* ∈ {5, 10, 15, 25} for other datasets. For the size of hidden states for encoder and decoder, we set *m = n* ∈ {16, 32, 64, 128}. The models are trained for 30 epochs with a batch size of 128. The initial learning rate is set as 0.001 and decays by 10% every 10 epochs.

To assess the performances of our model and comparison models, we adopt three evaluation metrics: mean absolute error (MAE), root mean square error (RMSE), and R squared (R2). MAE and RMSE are employed to measure the error between the predicted and observed values. R Squared (R2) is chosen as the indicator to measure the fitting effect of the model. The range of R2 is determined as (0,1). If R2 is close to 1, it means that the prediction accuracy of our model is high. MAE, RMSE, and R2 are defined as follows:

(16) $$\mathrm{MAE}\;=\;\frac{1}{N}\sum_{i=1}^{N}\left|y_{t}^{i}-{\hat{y}}_{t}^{i}\right|$$

(17) $$\mathrm{RMSE}\;=\sqrt{\frac{1}{N}\sum_{i=1}^{N}{\left(y_{t}^{i}-{\hat{y}}_{t}^{i}\right)}^{2}}$$

(18) $$R2=1-\frac{\sum_{i=1}^{N}\left|y_{t}^{i}-{\hat{y}}_{t}^{i}\right|}{\sum_{i=1}^{N\;}\left|y_{t}^{i}-\bar{y}\right|}$$

where ${\hat{y}}_{t}$ and $y_{t}$ are the predicted value and observed value at time *t*, $\bar{y}$ is the average value of the observed values, and *N* represents the number of samples. The scikit-learn package (https://scikit-learn.org/stable/index.html, accessed on 17 August 2022) provided support utilities of three evaluation metrics. Moreover, we implemented our model in the Pytorch framework (https://pytorch.org, accessed on 17 August 2022).

### 4.4. Experimental Results and Analyses

**Figure 4:**
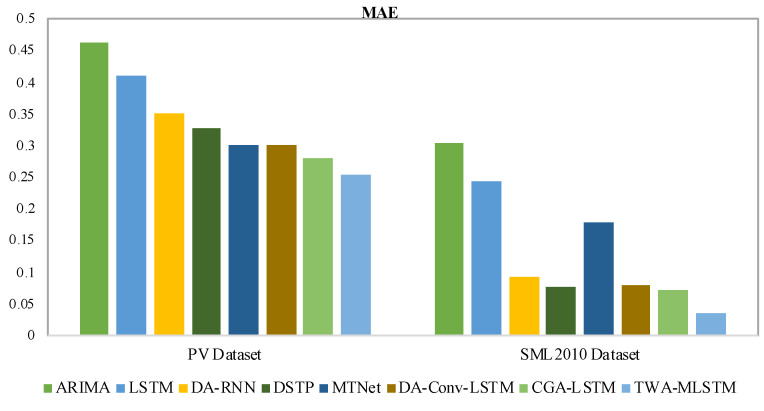
MAE values comparison of different models on PV power dataset and SML2010 dataset.

**Figure 5:**
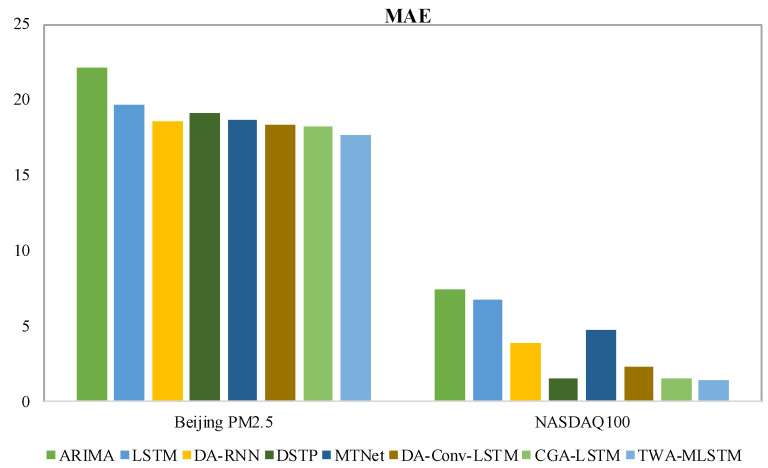
MAE values comparison of different models on Beijing PM2.5 dataset and NASDAQ100 stock dataset.

**Figure 6:**
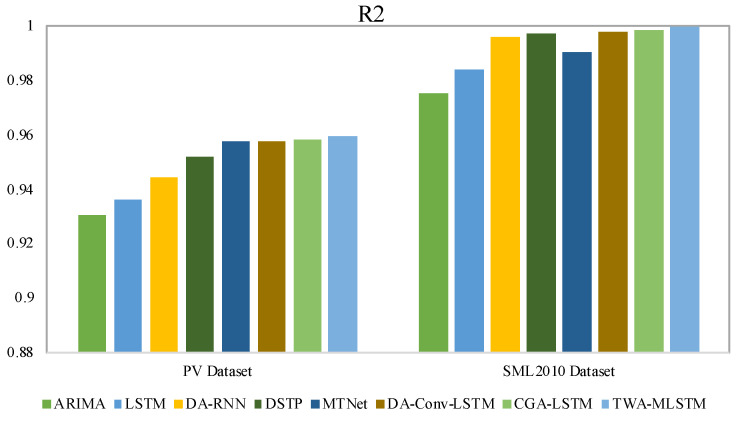
Fitting effect of different models on PV power dataset and SML2010 dataset.

**Figure 7:**
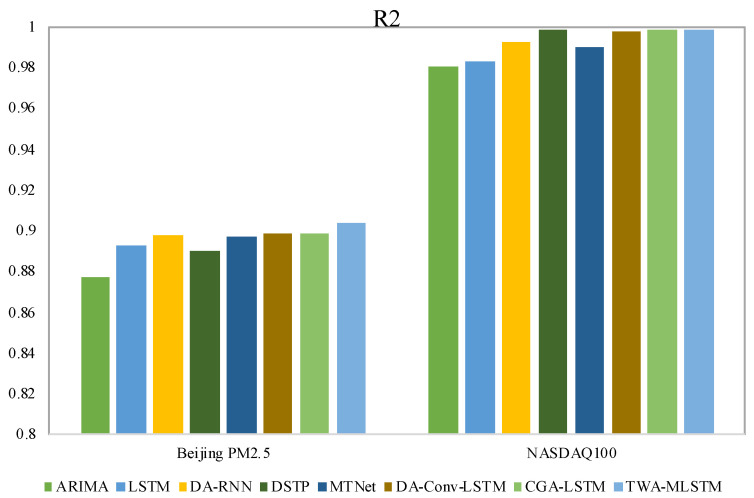
Fitting effect of different models on Beijing PM2.5 dataset and NASDAQ100 stock dataset.

In this section, we give the experimental results on four real-world datasets, evaluated with all three metrics, as shown in Table 2 and Table 3. The best results of each dataset are displayed in boldface. To clearly observe the difference in experimental results, we present the values of MAE and R2 with bar charts in Figure 4, Figure 5, Figure 6 and Figure 7. We observe that TWA-WDLSTM achieves better performance on all datasets.

As seen in Table 2 and Table 3, the MAE, RMSE, and R2 of ARIMA are higher than other contrast models and TWA-WDLSTM. TWA-WDLSTM shows 44.9%, 88.2%, 20.4% and 80.9% improvements over ARIMA in MAE on PV power, SML2010, Beijing PM2.5 and NASDAQ100 stock dataset, respectively. This suggests that ignoring exogenous factors can degrade model performance. Although LSTM achieves better performance than ARIMA, TWA-WDLSTM outperforms LSTM by 38.0%, 85.3%, 10.4% and 79.0% in MAE on four datasets, respectively. This is because the LSTM network focuses on extracting long-term dependencies of all time series rather than selecting relevant features.

DA-RNN, DSTP, MTNet, and DA-Conv-LSTM are the state-of-the-art models for multivariate time series, which pay more attention to obtaining the relevant variables in a time step and memorizing long-term dependencies among time series. Hence, their performance is better than ARIMA and LSTM. Nevertheless, these models exhibit different performances on four datasets. In detail, the DSTP model outperforms other state-of-the-art models on most tasks because the two-stage attention mechanism learns more stable spatial correlations. The performances of DA-RNN, MTNet, and DA-Conv-LSTM are comparable. The CGA-LSTM outperforms other contrast models because it nests a correlational attention into the graph attention mechanism to select the relevant variable.

For visual comparison, we display the MAE of comparison models and TWA-WDLSTM on four datasets in Figure 4 and Figure 5. In the comparison to state-of-the-art models, TWA-WDLSTM has the best performance on all datasets. For instance, the MAE value gained by TWA-WDLSTM (0.0358) is 61.6%, 53.1%, 79.9%, 55.4%, and 50.7% less than that of DA-RNN (0.0932), DSTP (0.0764), MTNet (0.1779), DA-Conv-LSTM (0.0803), and CGA-LSTM (0.0726) on SML2010 dataset. This suggests selecting the relevant variables in a temporal window to achieve accurate predictions. Moreover, WDLSTM employs the historical information of a temporal window to update the hidden state, which can capture very long term dependencies.

Figure 6 and Figure 7 visually present the fitting effects of different models on different datasets. We observe that TWA-WDLSTM has different fitting effects on four datasets. The data in the SML2010 and NASDAQ 100 stock datasets were more stable and controllable; hence, the R2 of TWA-WDLSTM is more than 0.999. Although the PV power dataset is periodic, no power is generated at night, which makes it more difficult to predict. However, TWA-WDLSTM still works better. TWA-WDLSTM shows inferiority on Beijing PM2.5 dataset but outperforms contrast models. This is because the randomness of Beijing PM2.5 data is stronger than other data.

### 4.5. Interpretability of Temporal Window Attention Mechanism

**Figure 8:**
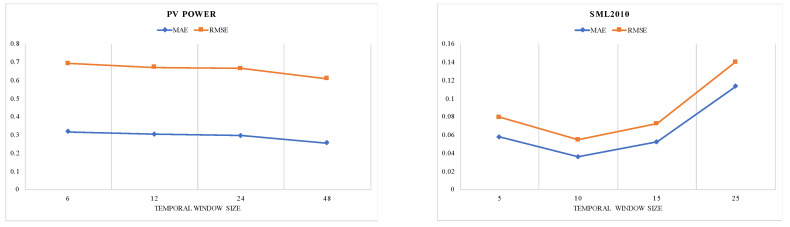
Influence of different temporal window size on model performance. When T = 48 on PV power dataset and T = 10 on SML2010 dataset, the model achieved the best performance.

**Figure 9:**
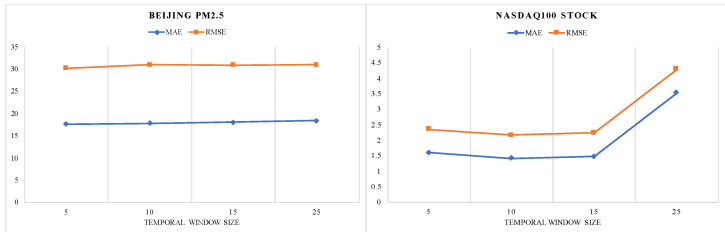
Influence of different temporal window size on TWA-WDLSTM performance. When *T* = 5 on Beijing PM2.5 dataset and *T* = 10 on ASDAQ100 stock dataset, the model achieved the best performance.

**Figure 10:**
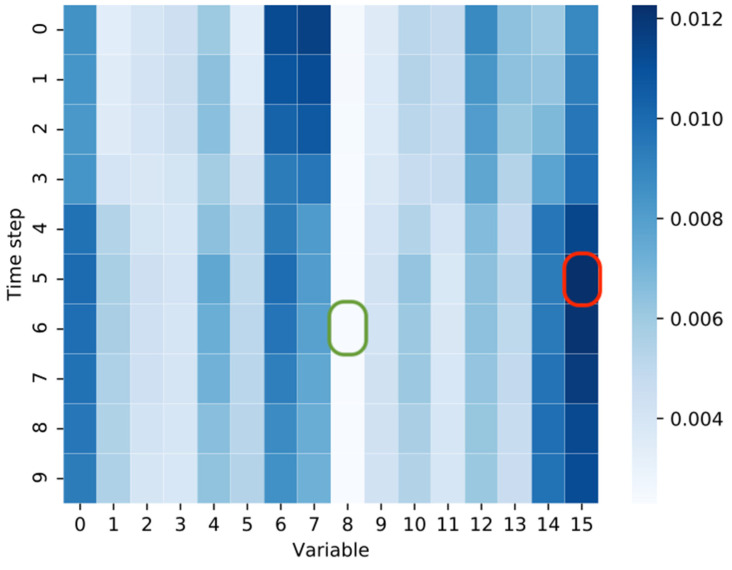
The attention weight matrix on SML2010 dataset. The red and green boxes highlight the maximum and minimum values, respectively.

The temporal window attention mechanism is employed to select the relevant variables in a temporal window for making the prediction. Hence, we verify its performance on different temporal window sizes using the grid search strategy. We plot the MAE and RMSE versus different temporal window sizes in Figure 8 and Figure 9. We observe that the minimum values of MAE, RMSE are gained when *T* = 48 on PV power dataset (*m = n* = 32), when *T* = 15 on SML2010 dataset (*m = n* = 32), when T = 5 on Beijing air dataset (*m = n* = 64), and when *T* = 10 on NASDAQ100 stock dataset (*m = n* = 128). To further investigate the temporal window attention mechanism, we visualize the weights distribution of the temporal window attention mechanism for the SML2010 dataset in Figure 10. The weights semantically represent the contribution of each variable in a temporal window to the future values. The more the corresponding variable contributes, the darker the color. For instance, the phenomenon can be clearly observed in that variable No. 15 at time step 5 (red box) exhibits a maximum contribution value of 0.0122, and variable No. 8 at time step 6 (green box) has a minimum contribution of about 0.0022. Moreover, multiple variables have different attention weights over different time steps. Specifically, the weights of variable No. 6 vary in the range of (0.0085, 0.0112). Variable No. 14 dynamically changes in the range of (0.0059, 0.0098). The above results illustrate that the temporal window attention mechanism successfully captures the relevant variable in a temporal window.

### 4.6. Evaluation on WDLSTM

To further evaluate the ability of WDLSTM to capture long-term dependencies, we compare its performance with LSTM on four real-world datasets that are described in Section 4.1, as presented in Table 4. Though the two networks share a similar intuition, WDLSTM outperforms LSTM because it benefits from the information flows of the temporal window. Specifically, WDLSTM shows 17.2%, 28.9%, 8.5% and 14.9% improvements beyond LSTM on MAE over four datasets. This is a significant outcome.

Moreover, TWA-WDLSTM shows 25.1%, 79.3%, 0.9%, and 75.2% improvements beyond WDLSTM on MAE over all datasets. The most striking result to emerge from the data is that the temporal window attention mechanism can adaptively extract the relevant variable to achieve accurate prediction.

## 5. Conclusions

In this paper, we propose the temporal window attention-based window-dependent long short-term memory network (TWA-WDLSTM), which consists of an encoder with a temporal window attention mechanism and a decoder, to make multivariate time series predictions. Extensive experiments on four real-world datasets strongly support our idea and show that TWA-WDLSTM outperforms the seven state-of-the-art models. The interpretation of the temporal window attention mechanism can further comprehend two-dimensional spatio-temporal patterns.

We summarize the significant advantages of TWA-WDLSTM as follows:

(1) In many actual cases, capturing the spatio-temporal correlations in multivariate time series is a challenge. However, most studies focus on capturing one-dimensional spatio-temporal correlations from a local perspective so that they could ignore some important information. The newly introduced temporal window attention mechanism can pick the important variables within a temporal window to capture two-dimensional spatio-temporal correlations from a global perspective.
(2) RNNs cannot memorize very long term information because they only summarize the information within a temporal window. To this end, we design WDLSTM as an encoder and decoder to enhance the learning ability of the long-term temporal dependencies.

The future works will further study whether the proposed model can be extended to solve the problem of long-term multivariate time series prediction by capturing more complex spatio-temporal patterns.

## Figures and Tables

**Table 1:** Hyperparameter settings.

Hyperparameter	Value
Training epochs	30
Batch size	128
hidden states	{16, 32, 64, 128}
Temporal window size	{6, 12, 24, 48}, {5, 10, 15, 25}
Initial Learning rate	0.001

**Table 2:** Experimental results on PV dataset and SML2010 dataset.

Dataset	PV Power			SML2010		
	MAE	RMSE	R2	MAE	RMSE	R2
ARIMA	0.4630	0.7951	0.9308	0.3033	0.3816	0.9754
LSTM	0.4119	0.7744	0.9365	0.2437	0.3080	0.9839
DA-RNN	0.3508	0.6356	0.9442	0.0932	0.1202	0.9962
DSTP	0.3280	0.6417	0.9523	0.0764	0.1106	0.9974
MTNet	0.3013	0.6251	0.9575	0.1779	0.2313	0.9903
DA-Conv-LSTM	0.3020	0.6218	0.9576	0.0803	0.1084	0.9980
CGA-LSTM	0.2796	0.6133	0.9582	0.0726	0.0959	0.9982
**TWA-WDLSTM**	**0.2552**	**0.6084**	**0.9594**	**0.0358**	**0.0546**	**0.9994**

**Table 3:** Experimental results on Beijing PM2.5 dataset and NASDAQ100 stock dataset.

Dataset	Beijing PM2.5			NASDAQ100 Stock		
	MAE	RMSE	R2	MAE	RMSE	R2
ARIMA	22.2014	34.2323	0.8771	7.4350	9.3034	0.9811
LSTM	19.7313	31.9733	0.8928	6.7567	8.7375	0.9833
DA-RNN	18.5451	31.2088	0.8982	3.8339	4.9586	0.9923
DSTP	19.1360	31.4934	0.8900	1.5583	2.2816	0.9988
MTNet	18.6874	30.1577	0.8974	4.7974	5.6801	0.9904
DA-Conv-LSTM	18.4088	31.0415	0.8990	2.2974	3.1040	0.9978
CGA-LSTM	18.2674	31.2421	0.8993	1.4952	2.2392	0.9990
**TWA-WDLSTM**	**17.6751**	**30.2252**	**0.9042**	**1.4189**	**2.1681**	**0.9990**

**Table 4:** Comparison result of LSTM and WDLSTM.

Dataset		LSTM	WDLSTM	TWA-WDLSTM
PV Power	MAE	0.4119	0.3409	**0.2552**
	RMSE	0.7744	0.6859	**0.6084**
	R2	0.9365	0.9485	**0.9594**
SML2010	MAE	0.2437	0.1732	**0.0358**
	RMSE	0.3080	0.2214	**0.0546**
	R2	0.9839	0.9917	**0.9994**
Beijing PM2.5	MAE	19.7313	18.038	**17.6751**
	RMSE	31.9733	30.5179	**30.2252**
	R2	0.8928	0.9024	**0.9042**
NASDAQ100 Stock	MAE	6.7567	5.7436	**1.4189**
	RMSE	8.7375	7.7110	**2.1681**
	R2	0.9833	0.9870	**0.9990**

## Data Availability

The datasets used and analyzed during the current study are available from the corresponding author on reasonable request.
